# MicroRNA-29a Attenuates Diabetic Glomerular Injury through Modulating Cannabinoid Receptor 1 Signaling

**DOI:** 10.3390/molecules24020264

**Published:** 2019-01-11

**Authors:** Chun-Wu Tung, Cheng Ho, Yung-Chien Hsu, Shun-Chen Huang, Ya-Hsueh Shih, Chun-Liang Lin

**Affiliations:** 1 Department of Nephrology, Chang Gung Memorial Hospital, Chiayi 61363, Taiwan; p122219@cgmh.org.tw (C.-W.T.); libra@cgmh.org.tw (Y.-C.H.); 2 Kidney and Diabetic Complications Research Team (KDCRT), Chang Gung Memorial Hospital, Chiayi 61363, Taiwan; hc1238@cgmh.org.tw; 3 Division of Endocrinology and Metabolism, Chang Gung Memorial Hospital, Chiayi 61363, Taiwan; 4 Department of Anatomic Pathology, Kaohsiung Chang Gung Memorial Hospital and Chang Gung University, College of Medicine, Kaohsiung 83301, Taiwan; shuang@cgmh.org.tw; 5 Kidney Research Center, Chang Gung Memorial Hospital, Taipei 10507, Taiwan; 6 College of Medicine, Chang Gung University, Taoyuan 33302, Taiwan; 7 Center for Shockwave Medicine and Tissue Engineering, Kaohsiung Chang Gung Memorial Hospital and Chang Gung University, College of Medicine, Kaohsiung 83301, Taiwan

**Keywords:** microRNA-29a, cannabinoid receptor type 1, PPAR-γ, diabetic nephropathy

## Abstract

Diabetic nephropathy often leads to end-stage renal disease and life-threatening morbidities. Simple control of risk factors is insufficient to prevent the progression of diabetic nephropathy, hence the need for discovering new treatments is of paramount importance. Recently, the dysregulation of microRNAs or the cannabinoid signaling pathway has been implicated in the pathogenesis of various renal tubulointerstitial fibrotic damages and thus novel therapeutic targets for chronic kidney diseases have emerged; however, the role of microRNAs or cannabinoid receptors on diabetes-induced glomerular injuries remains to be elucidated. In high-glucose-stressed renal mesangial cells, transfection of a miR-29a precursor sufficiently suppressed the mRNA and protein expressions of cannabinoid type 1 receptor (CB1R). Our data also revealed upregulated CB1R, interleukin-1β, interleukin-6, tumor necrosis factor-α, c-Jun, and type 4 collagen in the glomeruli of streptozotocin (STZ)-induced diabetic mice, whereas the expression of peroxisome proliferator-activated receptor-γ (PPAR-γ) was decreased. Importantly, using gain-of-function transgenic mice, we demonstrated that miR-29a acts as a negative regulator of CB1R, blocks the expressions of these proinflammatory and profibrogenic mediators, and attenuates renal hypertrophy. We also showed that overexpression of miR-29a restored PPAR-γ signaling in the renal glomeruli of diabetic animals. Collectively, our findings indicate that the interaction between miR-29a, CB1R, and PPAR-γ may play an important role in protecting diabetic renal glomeruli from fibrotic injuries.

## 1. Introduction

Diabetic nephropathy, which is the leading cause of end-stage renal disease globally, often leads to life-threatening morbidities [1]. During the progression of nephropathy, hyperglycemia or diabetic conditions induce inflammatory [2] and profibrotic [3] reactions, which are manifested pathologically as excessive deposition of extracellular matrix (ECM) in the glomerular mesangium, glomerular basement membrane thickening, and tubular atrophy, ultimately leading to glomerulosclerosis and renal fibrosis. Despite the significant effort that has been devoted to exploring the pathogenic factors, there are still no effective treatments for preventing or delaying the progression of diabetic kidney failure. This implies that there may be additional unknown mediators and mechanisms that need to be investigated.

The endocannabinoid system is essential to regulate a diversity of biological processes, including memory, appetite, energy metabolism, and immunity [4,5]. Upon ligand binding, the two Gi/o protein-coupled seven-transmembrane cannabinoid receptors, namely CB1R and CB2R, trigger intracellular signal cascades. The cannabinoid signaling reportedly regulates inflammatory and fibrotic reactions among various tissues [6,7,8,9,10]. Pharmacological inhibition or genetic deletion of CB1R has been shown to decrease fibrogenesis and improve survival of mice with bleomycin-induced pulmonary fibrosis [7]. Moreover, treatment with CB1R antagonist significantly decreases accumulation of hepatic myofibroblasts and expression of transforming growth factor-β1 (TGF-β1) in an acute model of matrix remodeling [10]. In contrary, recent data indicate that the activation of cannabinoid receptors dose-dependently decreases interleukin-6 (IL-6) and monocyte chemoattractant protein-1, as well as fibronectin, collagen 1, and α-smooth muscle actin levels in pancreatic stellate cells [8]. There are also contradictory results concerning the effects of cannabinoid receptors on renal tubulointerstitial fibrotic damage [11,12,13,14]. Targeted inhibition of CB1R or CB2R effectively reduces fibrosis in the kidneys of mice with unilateral ureteral obstruction [11,13]. In contrast, activation of CB2R signaling can alleviate tubulointerstitial fibrosis [12]. It is postulated that CB2R exerts antioxidant effects and lowers inflammation-driven fibrosis in some renal disease models. Although CB1R overexpression or CB2R deletion was shown to induce podocytopathy and proteinuria in vivo [15,16], the role of CB1R in diabetes-mediated glomerular injuries has not been fully elucidated.

MicroRNAs are short, highly conserved, and noncoding RNAs that regulate post-transcriptional gene expression via the interruption of protein translation and/or degradation of messenger RNAs (mRNAs). MicroRNAs are not only crucial in regulating renal development and homeostasis, but also play important roles in the progression of acute and/or chronic renal diseases [17,18]. Work from our laboratory and other groups has demonstrated the role of microRNAs in the pathogenesis of diabetic nephropathy, including the expression of proinflammatory cytokines, renal macrophage infiltration, podocyte dysfunction, and glomerular fibrosis [19,20,21]. One of the most abundant microRNAs in human tissues, the miR-29 family, is known to act as fibrotic regulators in several tissue types [22]. Previously, we have demonstrated that hyperglycemia reduces miR-29a expression in podocytes, which leads to increased expression of histone deacetylase 4 as well as subsequent deacetylation and ubiquitination of nephrin [20]. Overexpression of miR-29a in diabetic mice could efficiently stabilize nephrin, reduce proteinuria, and restore renal function. However, the role of miR-29a in diabetic glomerulosclerosis remains to be explored.

In this study, we sought to investigate whether miR-29a participates in diabetes-induced glomerular injuries and elucidate the relationship between miR-29a and CB1R signaling in vivo. Our findings revealed upregulation of proinflammatory and profibrotic gene expression, accompanied with activation of CB1R signaling in the glomerular mesangium of diabetic mice. Moreover, using gain-of-function transgenic mice, we showed that miR-29a is a negative regulator of CB1R. Mechanisms of how miR-29a modulates CB1R-regulated signaling in diabetic renal injuries are further discussed.

## 2. Results

### 2.1. MiR-29a Overexpression Significantly Reduces Diabetes-Induced Renal Hypertrophy

**Figure 1:**
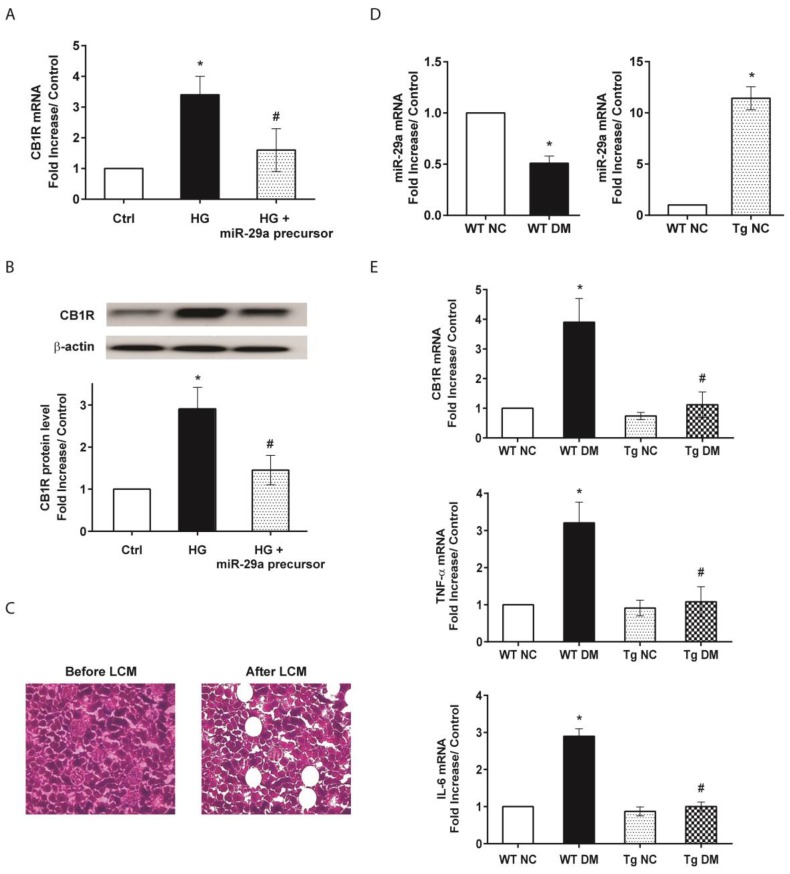
Influences of microRNA-29a (miR-29a) on the expressions of cannabinoid receptor type 1 (CB1R) and profibrotic genes in vitro and in glomeruli of diabetic mice. (**A**) Effects of high glucose (HG) and miR-29a precursor on CB1R expression in cultured renal mesangial cells by quantitative RT-PCR analysis. (**B**) Western blot analysis of CB1R protein in mesangial cells. Relative expression levels of CB1R protein quantified by densitometry are shown in the bottom panel. All experimental results from quantitative RT-PCR or Western blotting are presented as means ± standard error of the mean (SEM) calculated from three independent experiments. The symbol * indicates significant difference vs. the control group (*p* < 0.05); the symbol # indicates significant difference vs. the HG group (*p* < 0.05). (**C**) Isolation of glomerular components in renal tissues by laser capture microdissection (LCM). (**D**) mRNA expression of miR-29a in diabetic (left panel) and transgenic mice (right panel). (**E**) The mRNA expression levels of CB1R, tumor necrosis factor-α (TNF-α), and interleukin-6 (IL-6), normalized to that of β-actin, in glomeruli of wild-type normal control (WT NC), wild-type diabetic (WT DM), miR-29a transgenic normal control (Tg NC), and miR-29a transgenic diabetic mice were quantified by qRT-PCR (n = 6). The symbol * indicates significant difference vs. the WT NC group (*p* < 0.05); the symbol # indicates significant difference vs. the WT DM group (*p* < 0.05).

In order to explore whether miR-29a overexpression alleviated diabetic renal injuries, we created miR-29a transgenic mice expressing a human phosphoglycerate kinase (PGK) promoter-encoding miR-29a precursor (Figure 1). We also used streptozotocin (STZ)-treated mice in this study to further investigate the potential roles of miR-29a and CB1R signaling in diabetic glomerular damage. At 8 weeks after the successful induction of diabetes, STZ-induced diabetic mice showed higher levels of blood glucose, glycated hemoglobin A1c, and kidney-to-body weight ratios than their normal controls. Compared to diabetic mice, miR-29a overexpression did not affect glucose homeostasis. Of note, improvement of renal hypertrophy as evidenced by decreased kidney-to-body weight ratio was observed in miR-29a transgenic mice after induction of diabetes, which was consistent with our previous experimental results [20] (Table 1).

### 2.2. MiR-29a Modulates CB1R Expression and Ameliorates Renal Fibrotic Injuries

To clarify the effect of miR-29a on the modulation of CB1R signaling in the diabetic milieu, renal mesangial cells transiently transfected with a mature miR-29a precursor were used. Gain of miR-29a function significantly blocked high-glucose-upregulated CB1R mRNA and protein expression (Figure 1A,B). Using laser capture microdissection to harvest specific glomeruli (Figure 1C), quantitative reverse transcription-polymerase chain reaction (RT-PCR) analysis further confirmed that cells within glomeruli in diabetic kidneys displayed lower miR-29a (Figure 1D, left panel), but higher CB1R mRNA levels (Figure 1E) as compared to wild-type normal controls. To investigate whether miR-29a is involved in the regulation of the CB1R pathway in vivo, miR-29a transgenic mice (Figure 1D, right panel) with or without STZ treatment were used. Compared to wild-type diabetic mice, miR-29a transgenic mice had lower CB1R mRNA expressions in the glomeruli after STZ treatment (Figure 1E). These results suggest that miR-29a is involved in the control of specific CB1R signaling during the process of diabetic kidney injury.

**Figure 2:**
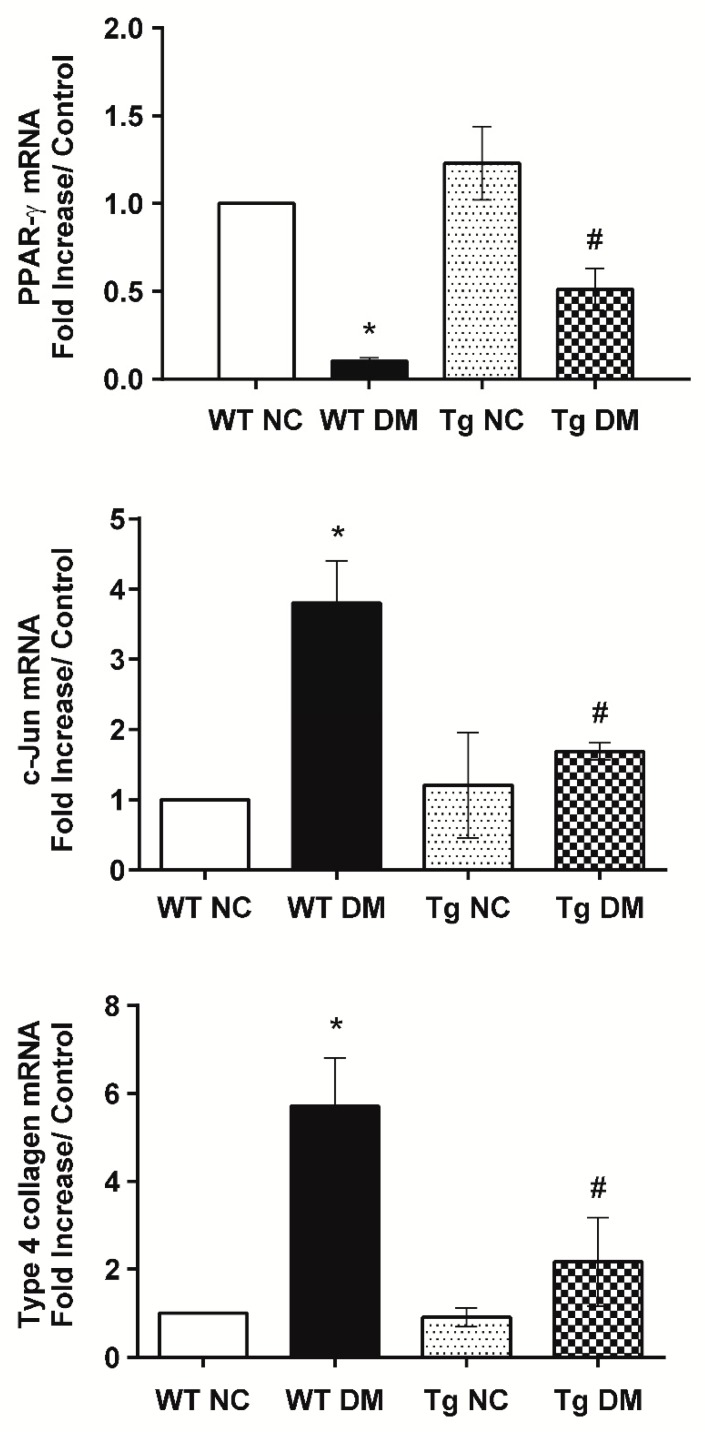
Reduction of c-Jun and type 4 collagen, but restoration of peroxisome proliferator-activated receptor-γ (PPAR-γ) mRNA levels in miR-29a transgenic mice after induction of diabetes. The mRNA expression levels of c-Jun, type 4 collagen, and PPAR-γ, normalized to that of β-actin, in microdissected glomeruli of study animals were quantified by qRT-PCR (n = 6). The symbol * indicates significant difference vs. the WT NC group (*p* < 0.05); the symbol # indicates significant difference vs. the WT DM group (*p* < 0.05).

**Figure 3:**
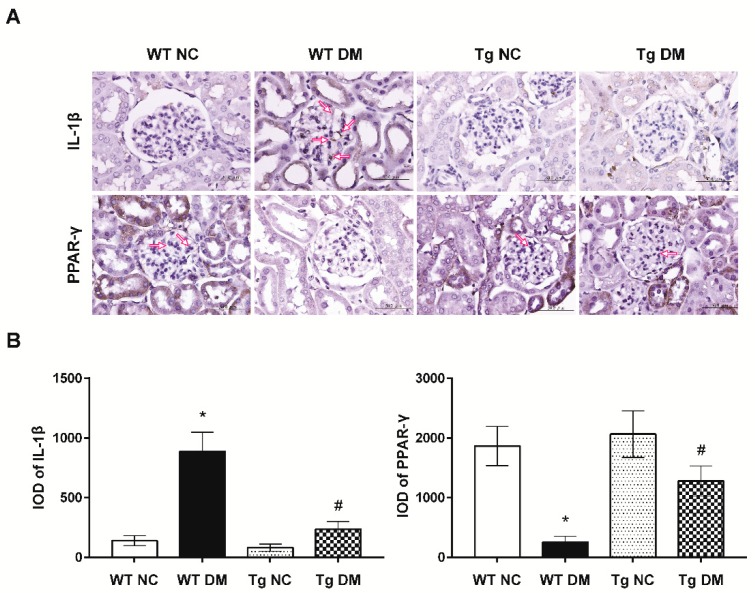
(**A**) Representative photographs of the immunohistochemical (IHC) staining for IL-1β and PPAR-γ in the renal cortex of study animals. Arrows indicate positive staining over mesangial cells or the mesangium. (**B**) The integrated optical density (IOD) of IL-1β and PPAR-γ IHC-stained materials were analyzed utilizing the Image Pro Plus software. IOD = optical intensity of positive cells × area of positive cells. Data from histomorphometric analysis are presented as means ± standard errors calculated from six independent experiments. The symbol * indicates significant difference vs. the WT NC group (*p* < 0.05); the symbol # indicates significant difference vs. the WT DM group (*p* < 0.05).

**Figure 4:**
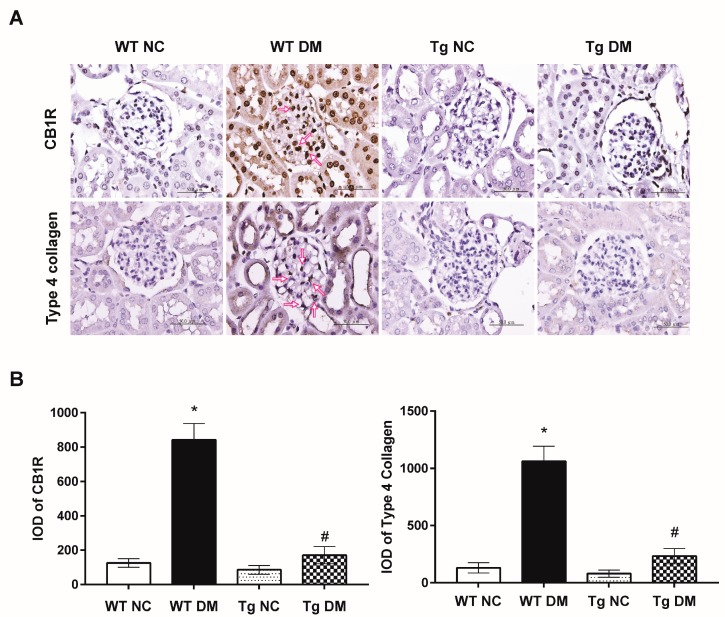
(**A**) Representative images of immunohistochemistry for CB1R and type 4 collagen in renal sections of study animals. Arrows indicate positive staining over mesangial cells or the mesangium. (**B**) The integrated optical density (IOD) of CB1R and type 4 collagen IHC-stained materials were analyzed . Data are presented as means ± standard errors calculated from six independent experiments. The symbol * indicates significant difference vs. the WT NC group (*p* < 0.05); the symbol # indicates significant difference vs. the WT DM group (*p* < 0.05).

Additionally, quantitative RT-PCR results demonstrated increased expression levels of inflammatory genes, tumor necrosis factor-α (TNF-α), and IL-6 mRNAs in diabetic mice (Figure 1E). In addition, kidney tissue samples from diabetic mice displayed higher mRNA levels of c-Jun and type 4 collagen as compared to those from the normal control group (Figure 2). Activation of c-Jun [23] or type 4 collagen [24,25] is known to be involved in the regulation of renal fibrosis and represents ECM deposition in diabetic nephropathy. When expression levels of inflammatory and fibrotic genes in renal glomeruli were evaluated, miR-29a transgenic mice treated with STZ had substantially lower TNF-α, IL-6, c-Jun, and type 4 collagen mRNA levels than wild-type STZ-treated mice (Figure 1E and Figure 2). Consistent with the quantitative RT-PCR analysis, immunohistochemical staining and histomorphometric analysis showed that miR-29a transgenic mice following STZ treatment expressed significantly lower interleukin-1β (IL-1β) (Figure 3) and type 4 collagen (Figure 4) in the glomerular mesangium than wild-type mice after induction of diabetes.

### 2.3. MiR-29a Attenuates CB1R but Restores Peroxisome Proliferator-Activated Receptor-γ (PPAR-γ) Expressions in Diabetic Animals

To determine whether miR-29a modulates CB1R signaling in diabetic nephropathy, we examined mRNA levels of CB1R in the renal glomeruli of miR-29a transgenic mice with and without induction of diabetes. Compared to wild-type diabetic mice, we found that miR-29a transgenic mice had lower CB1R expression levels following STZ treatment (Figure 1E). Consistently, high-power field microscopic analysis of glomeruli also revealed that renal sections from miR-29a transgenic group displayed less positive IHC staining for CB1R than those from wild-type mice after induction of diabetes (Figure 4). Emerging evidence has shown the protective effects of PPAR-γ against diabetic kidney disease [26,27]; our quantitative RT-PCR and immunohistochemical studies (Figure 2 and Figure 3) showed that overexpression of miR-29a significantly reversed the attenuated PPAR-γ expression in diabetic mice.

## 3. Discussion

The role of microRNAs in kidney diseases has been investigated intensively in the past few years, and much attention has been focused on the miR-29 family (miR-29a, miR-29b, and miR-29c) for its direct targeting of ECM genes. In high-glucose-treated human proximal tubular HK-2 cells, downregulated miR-29 variants increase the expression of collagen type 4 [28]. In addition to renal tubules, the phenomenon of reduced expression of the miR-29a/b/c family increasing production of ECM proteins was observed in TGF-β1-stimulated primary mesangial cells and podocytes [29]. Conversely, miR-29c was shown to be upregulated and to promote inflammatory responses and induce ECM protein accumulation and podocyte apoptosis under similar diabetic conditions [30,31]. Due to the pleiotropic roles and actions of the miR-29 family in different cell types and disease conditions, the detailed function and regulation of miR-29a in the pathogenesis of diabetic glomerular injuries need to be further elucidated. Previously, our in situ hybridization data have showed that miR-29a expression was significantly reduced in the glomeruli of STZ-induced diabetic mice [20,21]. We have demonstrated the importance of miR-29a regulation in the maintenance of the integrity of podocyte ultrastructures, such as nephrin and podocin in diabetic kidneys [20]. More recently, we also showed the important roles of miR-29a and CB1R signaling in the pathogenesis of glomerulosclerosis and renal fibrosis. Periodic acid–Schiff (PAS) stain showed that exogenous miR-29a expression in diabetic mice effectively decreased the mesangial matrix expansion [21]. Additionally, CB1R transgenic mice strongly displayed PAS and fibronectin staining in the glomerular mesangium compared to wild-type mice. Our immunofluorescence experiments also showed colocalization of enhanced CB1R signaling and smooth muscle actin/Thy1-positive mesangial cells in diabetic mice [32]. Treatment with CB1R antisense oligonucleotides significantly decreased the mRNA levels of profibrotic genes in high-glucose-stressed mesangial cells and reduced the PAS stain intensity in STZ-treated mice [32]. In this study, we further confirmed the protective effects of miR-29a against inflammation and fibrosis in diabetes-induced glomerulosclerosis through modulating CB1R signaling. Of note, a recent report disclosed that a newly introduced antidiabetic drug, linagliptin, a dipeptidyl peptidase-4 (DPP-4) inhibitor, ameliorates renal fibrosis in diabetic mice via the restoration of miR-29 variants [33]. These new approaches targeting miR-29a and other potential microRNAs may offer new treatments for patients with diabetic nephropathy.

In this study, we observed that the gain of miR-29a function is associated with the reversal of PPAR-γ expression (Figure 2 and Figure 4). A meta-analysis of 2860 diabetic patients convincingly demonstrated the significant efficacy of PPAR-γ agonists on reducing proteinuria [34]. Recently, mounting evidence has suggested the importance of the microRNA-dependent regulation of PPAR-γ in metabolic and malignant diseases [35]. Upregulated miR-27a in hyperglycemia-stimulated renal tubular cells and STZ-induced diabetic rats was shown to trigger PPAR-γ downregulation, which in turn contributes to unfavorable renal outcomes [36]. Besides, whole-transcriptome sequencing data revealed that loss of miR-29 function increases the mRNA and protein levels of the deacetylase sirtuin 1, which is a critical regulator of PPAR-γ signaling [37].

On the other hand, overexpression of miR-29a significantly reversed CB1R upregulation in vitro and in vivo (Figure 1 and Figure 4). Epigenetic modifications have been shown to directly regulate the endocannabinoid system in a variety of cell types [38]. Möhnle et al. provided evidence that cannabinoid receptors are subject to microRNA regulation in cultured human cardiomyocytes [39]. CB1R mRNA and protein levels were significantly reduced in cardiac cells transiently transfected with pre-miR-494. Additionally, miR-139 was shown to impair the hippocampus-dependent learning and memory functions by targeting CB2R in an accelerated-aging animal model [40]. Immunohistochemical analysis showed that overexpression of miR-139 resulted in a decrease in the number of CB2R-positive nuclei in the hippocampus. In this manuscript, we describe the regulation of microRNA on a cannabinoid receptor in renal tissues.

**Figure 5:**
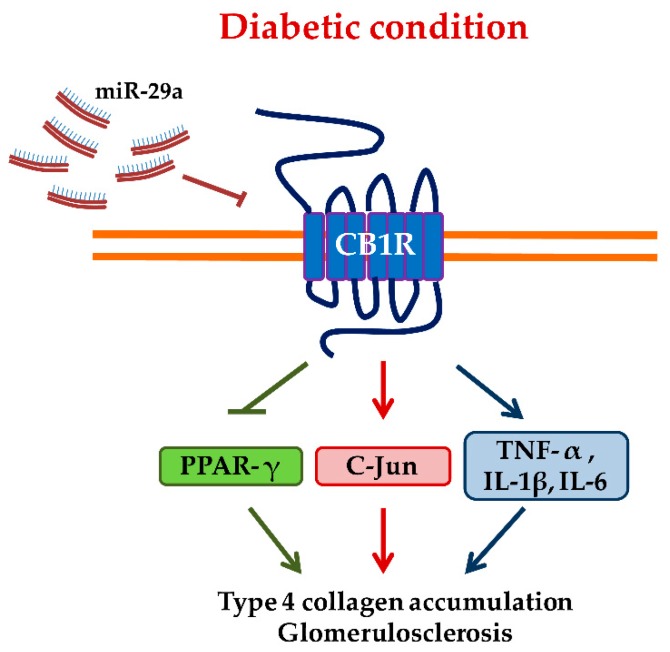
Schematic illustration of how miR-29a modulates CB1R-regulated signaling in diabetic renal injuries. Abbreviations: CB1R, cannabinoid type 1 receptor; PPAR-γ, proliferator-activated receptor-γ; TNF-α, tumor necrosis factor-α; IL, interleukin.

Growing evidence also suggests that endocannabinoids are important regulators of PPAR-γ [41]. Data from our previous experiments have reported the reciprocal regulation between CB1R and PPAR-γ in diabetic rats [32]. Breakdown of CB1R by antisense oligonucleotides or using a selective CB1R antagonist was shown to restore PPAR-γ signaling. In addition, treatment with rosiglitazone, a PPAR-γ agonist, effectively reduced the upregulated CB1R signaling, inflammation, and glomerular fibrosis in diabetic animals [32]. Hence, we hypothesize that miR-29a reverses CB1R-mediated PPAR-γ signaling to protect diabetic kidneys from the fibrotic process (Figure 5). To our knowledge, this is the first report showing the regulation of CB1R and PPAR-γ signaling by microRNAs in diabetic nephropathy in vivo.

Moreover, we presented that upregulated CB1R (Figure 1 and Figure 4) is associated with increased expression of c-Jun mRNA in diabetic renal glomeruli. Stimulation of CB1R has been shown to activate c-Jun N-terminal kinase (JNK), which can regulate nuclear transcription factors [42]. The use of an agonist acting on CB1R is sufficient to increase c-Jun expression in mouse neuroblastoma cells [43]. Additionally, pharmacological antagonism of CB1R has been shown to inhibit the nuclear translocation and DNA binding of c-Jun and c-Fos in receptor activator of nuclear factor kappa-B ligand (RANKL)-generated mouse osteoclasts [44]. We have also previously reported that knockdown of CB1R signaling effectively attenuates the hyperglycemia-induced upregulation of c-Jun, TGF-β1, IL-6, and TNF-α in vitro and in vivo [32]. These evidences imply that c-Jun and some proinflammatory genes may act as a downstream effector of CB1R signaling (Figure 5). Taken together, miR-29a plays a pivotal role in the pathogenesis of diabetic glomerulosclerosis. We propose that overexpression of miR-29a may effectively repress CB1R-mediated inflammation, ECM accumulation, and reduction of PPAR-γ signaling (Figure 5).

## 4. Materials and Methods

### 4.1. In Vitro High-Glucose-Treated Mesangial Cell Cultures

Normal rat mesangial cells (NRK-52E, CRL-2573^TM^) were obtained from the American Type Culture Center, Manassas, VA, USA). Cells (1 × 10^6^ cells/well, 6-well plates) were incubated in Dulbecco’s modified Eagle’s medium (DMEM) supplemented with 10% fetal bovine serum (Gibco, Carlsbad, CA, USA) and 35 mM d-glucose (high glucose treatment) or 35 mM d-mannose (osmolarity control) for 48 h.

### 4.2. Transfection of MicroRNA-29a (miR-29a) Precursor

To examine the effect of miR-29a on CB1R expression, a double-stranded RNA oligonucleotide (Pre-miR-29a^TM^ miRNA Precursor Molecules, Applied Biosystems-Ambio Inc., Austin, TX, USA) was used. Renal mesangial cells (3 × 10^5^ cells/well, six-well plates) were cultured in DMEM and 10% fetal bovine serum until 70–80% confluency and then were transiently transfected with the miR-29a precursor using Lipofectamine 2000 Transfection Reagent (Invitrogen, Carlsbad, CA, USA) according to the manufacturer’s instructions.

### 4.3. Protein Extraction and Western Blotting Analysis

Total cellular proteins were extracted from renal cells as described previously [45]. Equal aliquots of lysates were fractionated on 8–12% acrylamide gel and the blots were probed with primary antibodies against CB1R (Abcam, Cambridge, UK), followed by horseradish peroxidase-conjugated IgG (Santa Cruz Biotechnology, Dallas, TX, USA) as the secondary antibody, and visualized by chemiluminescence. These membranes were stripped and then reprobed with β-actin (Cell Signaling Technology, Beverly, MA, USA) with the same procedures to show the equal loading. The relative intensities of immunoblot signals were measured by densitometry using Image-Pro Plus 6.0 image analysis software (SNAP-Pro c.f. Digital kit; Media Cybernetics Inc., Silver Spring, MD, USA).

### 4.4. MiR-29a Transgenic Mice

MiR-29a transgenic mice (FVB/miR-29a^Tg^) were generated and maintained as described previously [20,21]. Briefly, the PGK promoter and full-length miR-29a precursor sequences were cloned from a cDNA library by PCR. The cDNAs were then inserted into the pUSE empty expression vector, and the linear PGK-miR-29a-BGH poly-A cDNAs were cloned. The designed miR-29a-containing DNA constructs were then transferred into fertilized eggs from FVB/N mice. The eggs were further transferred into Crl:CD1 (ICR) foster mothers. Transgenic mice were bred in a specific pathogen-free condition and genotyped by PCR using specific primers (forward: 5′-GAGGATCCCCTCAAGGATACCAAGGGATGAAT-3′ and reverse: 5′-CTTCTAGAAGGAGTGTTTCTAGGTATCCGTCA-3′). A total of 24 mice were randomly subdivided into four groups, including the vehicle-treated wild-type normal control group (n = 6), the streptozotocin (STZ)-induced wild-type diabetic group (n = 6), the vehicle-treated transgenic control group (n = 6), and the STZ-induced transgenic diabetic group (n = 6). All protocols for animal use and experiments were approved by the Institutional Animal Care and Use Committee of Chang Gung Memorial Hospital (Approval No.: 2014062402) and were performed in accordance with the Animal Protection Law by the Council of Agriculture, Executive Yuan (R.O.C.) and the guidelines of the National Research Council (USA) for the care and use of laboratory animals.

### 4.5. Streptozotocin (STZ)-Induced Diabetic Animal Models

Four-month-old male wild-type or miR-29a transgenic FVB mice (BioLasco Biotechnology Co., Taipei, Taiwan) were injected intraperitoneally with 50 mg/kg STZ for five consecutive days. Two weeks after injection, animals with fasting blood glucose of 200–300 mg/dL were considered diabetic and selected for further studies. For equalization of glycemic control, intermediate-acting insulin (Monotard; Novo Nordisk A/S, Bagsvaerd, Denmark) of 1 to 2 units/kg was administered subcutaneously once a day until the mice were sacrificed. Diabetic or normal animals were later euthanized by an overdose of chloral hydrate at 8 weeks after induction of diabetes, and renal tissues were dissected for studies.

### 4.6. Blood Biochemistry and Measurement of Kidney Weight

Serum levels of glucose (Dade Behring Inc., Newark, NJ, USA) and glycated hemoglobin A1c (HbA1c, Primus Diagnostics, Trinity Biotech Co., Kansas City, MO, USA) were determined according to the manufacturer’s instructions. Mice kidneys were removed at termination and weighed. The kidney-to-body weight ratio was obtained and reported with the unit of mg/g.

### 4.7. Preparation and Laser Capture Microdissection (LCM) of Kidney Tissues

After adequate perfusion with phosphate-based saline (PBS) solutions, both kidneys were harvested and immediately frozen with optimal cutting temperature (OCT) compound for LCM studies. Freshly frozen kidney tissues were sliced into 4-μm thick sections with a cryostat. Laser capture microdissection were performed under RNase-free conditions as described previously [46]. Briefly, renal sections were covered with LCM transfer film (CapSure TF-100; Arcturus Engineering, Inc., Mountain View, CA, USA), and specific glomerular portions of the histologic section were captured using a VERITAS™ laser-captured dissection system (Arcturus Bioscience Inc., Sunnyvale, CA, USA) according to the manufacturer’s instructions. Two hundred glomeruli from 6 sections of each animal in each group were dissected to extract the total RNA for subsequent quantitative RT-PCR analysis.

### 4.8. Quantitative Reverse Transcription-PCR (qRT-PCR)

Total RNA was extracted from isolated glomerular tissues or mesangial cells by using QIAzol lysis reagent (Qiagen, Valencia, CA, USA) according to the manufacturer’s instructions. One microgram of total RNA was reverse-transcribed into cDNA with the ReverAid^TM^ M-MuLV reverse transcriptase (Fermentas, Glen Burnie, MD, USA). Twenty-five microliters of qPCR mixture containing cDNA templates (equivalent to 20 ng of total RNA), 2.5 μM of each forward and reverse primers, and 2X iQ^TM^ SYBR Green Supermix (Bio-Rad, Hercules, CA, USA) were amplified in an iCycler iQ Real-time PCR Detection System (Bio-Rad Laboratories, Hercules, CA, USA), with an initial melting step at 95 °C for 5 min, followed by 35 cycles of denaturation at 94 °C for 15 s, annealing at 52 °C for 20 s, and extension at 72 °C for 30 s. The following gene-specific PCR primers were used in this study (sense and antisense): 5′-TGCTTGATGTGGACTATTGC-3′ and 5′-TGAGGTGTGAATGATGATGC-3′ for CB1R; 5′-AGATGTGGAACTGGCAGAGG-3′ and 5′-CCCATTTGGGAACTTCTCCT-3′ for TNF-α; 5′-CCGGAGAGGAGACTTCACAG-3′ and 5′-ACAGTGCATCATCGCTGTTC-3′ for IL-6; 5′-CCAAAGCCTGAGCCCAGA-3′ and 5′-GCACCACTCCCATGGCAT-3′ for PPAR-γ; 5′-CAGGTGGCACAGCTTAAACA-3′ and 5′-CGCAACCAGTCAAGTTCTCA-3′ for c-Jun; 5′-GACCCCGAGGTTAGGAAGG-3′ and 5′-CACTCGGTCCATGATCCCA-3′ for type 4 collagen; 5′-CGCCAACCGCGAGAAGAT-3′ and 5′-CGTCACCGGAGTCCATCA-3′ for β-actin. All quantitative RT-PCR experiments were performed in duplicate from at least six independent experiments. The relative gene expression was calculated as described previously [47].

### 4.9. Immunohistochemistry

After being adequately perfused with PBS solutions, harvested kidneys were fixed in 4% PBS-buffered formaldehyde, embedded in paraffin, and then sliced longitudinally into 4-μm thick sections. Renal specimens were immunostained with primary antibodies against CB1R (BioWorld Tech., St. Louis Park, MN, USA), type 4 collagen, IL-1β, and PPAR-γ (Abcam, Cambridge, UK). Immunoreactivity in sections was detected using a horseradish peroxidase-3,3′-diaminobenzidine kit (SuperPicture™ Kit, Invitrogen, Carlsbad, CA, USA). Ten glomeruli in each section were randomly selected for microscopy under 200× magnification (Carl Zeiss, Gottingen, Germany). Six regions within renal glomeruli from three sections obtained from six mice were observed. The areas of positive immunolabeled regions were quantified by using Image-Pro^®^ Plus image analysis software (Media Cybernetics, Silver Spring, MD, USA).

### 4.10. Statistical Analysis

All values present in the study were expressed as means ± standard errors. One-way analysis of variance followed by the Bonferroni post-hoc test was performed to compare the differences among groups. *p* < 0.05 was considered to be statistically significant.

## 5. Conclusions

Overall, our studies identify miR-29a as an important and novel regulator of glomerular fibrosis in diabetic nephropathy. Overexpressing miR-29a reduces the expression of CB1R, as well as inflammatory and fibrogenic genes. Therapies aimed at activating miR-29a or inhibiting CB1R signaling deserve attention as potential treatments for diabetic nephropathy.

## Figures and Tables

**Table 1:** Biochemical properties and the kidney-to-total body weight ratios of study animals.

	WT NC	WT DM	Tg NC	Tg DM
Blood glucose (mg/dL)	140 ± 11.1	439.4 ± 3.6 *	139 ± 10.1	470.3 ± 2.6 *
HbA1c (%)	4.2 ± 0.3	8.1 ± 1.1 *	4.3 ± 0.9	8.3 ± 1.3 *
Kidney weight/body weight (mg/g)	9 ± 4	13 ± 2.1 *	8.7 ± 2.3	9.3 ± 3.4 ^#^

Data are expressed as means ± standard errors calculated from six animals in each group. Abbreviations: WT: wild-type; NC: normal control; DM: diabetes mellitus; Tg: miR-29a transgenic. The symbol * indicates significant difference vs. the wild-type normal (WT NC) group (*p* < 0.05); the symbol # indicates significant difference vs. the wild-type diabetic (WT DM) group (*p* < 0.05).

## References

[1] Jha V., Garcia-Garcia G., Iseki K., Li Z., Naicker S., Plattner B., Saran R., Wang A.Y., Yang C.W. (2013). Chronic kidney disease: Global dimension and perspectives. Lancet.

[2] Kanasaki K., Taduri G., Koya D. (2013). Diabetic nephropathy: The role of inflammation in fibroblast activation and kidney fibrosis. Front. Endocrinol..

[3] Umanath K., Lewis J.B. (2018). Update on Diabetic Nephropathy: Core Curriculum 2018. Am. J. Kidney Dis..

[4] Busquets-Garcia A., Gomis-Gonzalez M., Srivastava R.K., Cutando L., Ortega-Alvaro A., Ruehle S., Remmers F., Bindila L., Bellocchio L., Marsicano G. (2016). Peripheral and central CB1 cannabinoid receptors control stress-induced impairment of memory consolidation. Proc. Natl. Acad. Sci. USA.

[5] Liu L.Y., Alexa K., Cortes M., Schatzman-Bone S., Kim A.J., Mukhopadhyay B., Cinar R., Kunos G., North T.E., Goessling W. (2016). Cannabinoid receptor signaling regulates liver development and metabolism. Development.

[6] Zurier R.B., Burstein S.H. (2016). Cannabinoids, inflammation, and fibrosis. FASEB J..

[7] Cinar R., Gochuico B.R., Iyer M.R., Jourdan T., Yokoyama T., Park J.K., Coffey N.J., Pri-Chen H., Szanda G., Liu Z. (2017). Cannabinoid CB1 receptor overactivity contributes to the pathogenesis of idiopathic pulmonary fibrosis. JCI Insight.

[8] Michalski C.W., Maier M., Erkan M., Sauliunaite D., Bergmann F., Pacher P., Batkai S., Giese N.A., Giese T. (2008). Cannabinoids Reduce Markers of Inflammation and Fibrosis in Pancreatic Stellate Cells. PLoS ONE.

[9] Servettaz A., Kavian N., Nicco C., Deveaux V., Chereau C., Wang A., Zimmer A., Lotersztajn S., Weill B., Batteux F. (2010). Targeting the cannabinoid pathway limits the development of fibrosis and autoimmunity in a mouse model of systemic sclerosis. Am. J. Pathol..

[10] Teixeira-Clerc F., Julien B., Grenard P., Tran Van Nhieu J., Deveaux V., Li L., Serriere-Lanneau V., Ledent C., Mallat A., Lotersztajn S. (2006). CB1 cannabinoid receptor antagonism: A new strategy for the treatment of liver fibrosis. Nat. Med..

[11] Lecru L., Desterke C., Grassin-Delyle S., Chatziantoniou C., Vandermeersch S., Devocelle A., Vernochet A., Ivanovski N., Ledent C., Ferlicot S. (2015). Cannabinoid receptor 1 is a major mediator of renal fibrosis. Kidney Int..

[12] Tang M., Cao X., Zhang K., Li Y., Zheng Q.Y., Li G.Q., He Q.H., Li S.J., Xu G.L., Zhang K.Q. (2018). Celastrol alleviates renal fibrosis by upregulating cannabinoid receptor 2 expression. Cell Death Dis..

[13] Zhou L., Zhou S., Yang P., Tian Y., Feng Z., Xie X.-Q., Liu Y. (2018). Targeted inhibition of the type 2 cannabinoid receptor is a novel approach to reduce renal fibrosis. Kidney Int..

[14] Barutta F., Bruno G., Mastrocola R., Bellini S., Gruden G. (2018). The role of cannabinoid signaling in acute and chronic kidney diseases. Kidney Int..

[15] Hsu Y.C., Lei C.C., Shih Y.H., Ho C., Lin C.L. (2015). Induction of proteinuria by cannabinoid receptors 1 signaling activation in CB1 transgenic mice. Am. J. Med. Sci..

[16] Barutta F., Grimaldi S., Franco I., Bellini S., Gambino R., Pinach S., Corbelli A., Bruno G., Rastaldi M.P., Aveta T. (2014). Deficiency of cannabinoid receptor of type 2 worsens renal functional and structural abnormalities in streptozotocin-induced diabetic mice. Kidney Int..

[17] Wei Q., Mi Q.-S., Dong Z. (2013). The regulation and function of micrornas in kidney diseases. IUBMB Life.

[18] Ichii O., Horino T. (2018). MicroRNAs associated with the development of kidney diseases in humans and animals. J. Toxicol. Pathol..

[19] Bhatt K., Lanting L.L., Jia Y., Yadav S., Reddy M.A., Magilnick N., Boldin M., Natarajan R. (2015). Anti-Inflammatory Role of MicroRNA-146a in the Pathogenesis of Diabetic Nephropathy. J. Am. Soc. Nephrol..

[20] Lin C.L., Lee P.H., Hsu Y.C., Lei C.C., Ko J.Y., Chuang P.C., Huang Y.T., Wang S.Y., Wu S.L., Chen Y.S. (2014). MicroRNA-29a promotion of nephrin acetylation ameliorates hyperglycemia-induced podocyte dysfunction. J. Am. Soc. Nephrol..

[21] Hsu Y.C., Chang P.J., Ho C., Huang Y.T., Shih Y.H., Wang C.J., Lin C.L. (2016). Protective effects of miR-29a on diabetic glomerular dysfunction by modulation of DKK1/Wnt/beta-catenin signaling. Sci. Rep..

[22] Kriegel A.J., Liu Y., Fang Y., Ding X., Liang M. (2012). The miR-29 family: Genomics, cell biology, and relevance to renal and cardiovascular injury. Physiol. Genomics.

[23] De Borst M.H., Prakash J., Melenhorst W.B., van den Heuvel M.C., Kok R.J., Navis G., van Goor H. (2007). Glomerular and tubular induction of the transcription factor c-Jun in human renal disease. J. Pathol..

[24] Adler S.G., Feld S., Striker L., Striker G., LaPage J., Esposito C., Aboulhosn J., Barba L., Cha D.R., Nast C.C. (2000). Glomerular type IV collagen in patients with diabetic nephropathy with and without additional glomerular disease. Kidney Int..

[25] Umezono T., Toyoda M., Kato M., Miyauchi M., Kimura M., Maruyama M., Honma M., Yagame M., Suzuki D. (2006). Glomerular expression of CTGF, TGF-beta 1 and type IV collagen in diabetic nephropathy. J. Nephrol..

[26] Balakumar P., Arora M.K., Singh M. (2009). Emerging role of PPAR ligands in the management of diabetic nephropathy. Pharmacol. Res..

[27] Yang J., Zhou Y., Guan Y. (2012). PPARgamma as a therapeutic target in diabetic nephropathy and other renal diseases. Curr. Opin. Nephrol. Hypertens..

[28] Du B., Ma L.M., Huang M.B., Zhou H., Huang H.L., Shao P., Chen Y.Q., Qu L.H. (2010). High glucose down-regulates miR-29a to increase collagen IV production in HK-2 cells. FEBS Lett..

[29] Wang B., Komers R., Carew R., Winbanks C.E., Xu B., Herman-Edelstein M., Koh P., Thomas M., Jandeleit-Dahm K., Gregorevic P. (2012). Suppression of microRNA-29 expression by TGF-beta1 promotes collagen expression and renal fibrosis. J. Am. Soc. Nephrol..

[30] Guo J., Li J., Zhao J., Yang S., Wang L., Cheng G., Liu D., Xiao J., Liu Z., Zhao Z. (2017). MiRNA-29c regulates the expression of inflammatory cytokines in diabetic nephropathy by targeting tristetraprolin. Sci. Rep..

[31] Long J., Wang Y., Wang W., Chang B.H., Danesh F.R. (2011). MicroRNA-29c is a signature microRNA under high glucose conditions that targets Sprouty homolog 1, and its in vivo knockdown prevents progression of diabetic nephropathy. J. Biol. Chem..

[32] Lin C.L., Hsu Y.C., Lee P.H., Lei C.C., Wang J.Y., Huang Y.T., Wang S.Y., Wang F.S. (2014). Cannabinoid receptor 1 disturbance of PPARgamma2 augments hyperglycemia induction of mesangial inflammation and fibrosis in renal glomeruli. J. Mol. Med..

[33] Kanasaki K., Shi S., Kanasaki M., He J., Nagai T., Nakamura Y., Ishigaki Y., Kitada M., Srivastava S.P., Koya D. (2014). Linagliptin-mediated DPP-4 inhibition ameliorates kidney fibrosis in streptozotocin-induced diabetic mice by inhibiting endothelial-to-mesenchymal transition in a therapeutic regimen. Diabetes.

[34] Sarafidis P.A., Stafylas P.C., Georgianos P.I., Saratzis A.N., Lasaridis A.N. (2010). Effect of thiazolidinediones on albuminuria and proteinuria in diabetes: A meta-analysis. Am. J. Kidney Dis..

[35] Portius D., Sobolewski C., Foti M. (2017). MicroRNAs-Dependent Regulation of PPARs in Metabolic Diseases and Cancers. PPAR Res..

[36] Hou X., Tian J., Geng J., Li X., Tang X., Zhang J., Bai X. (2016). MicroRNA-27a promotes renal tubulointerstitial fibrosis via suppressing PPARgamma pathway in diabetic nephropathy. Oncotarget.

[37] Kurtz C.L., Fannin E.E., Toth C.L., Pearson D.S., Vickers K.C., Sethupathy P. (2015). Inhibition of miR-29 has a significant lipid-lowering benefit through suppression of lipogenic programs in liver. Sci. Rep..

[38] D’Addario C., Di Francesco A., Pucci M., Finazzi Agrò A., Maccarrone M. (2013). Epigenetic mechanisms and endocannabinoid signalling. FEBS J..

[39] Mohnle P., Schutz S.V., Schmidt M., Hinske C., Hubner M., Heyn J., Beiras-Fernandez A., Kreth S. (2014). MicroRNA-665 is involved in the regulation of the expression of the cardioprotective cannabinoid receptor CB2 in patients with severe heart failure. Biochem. Biophys. Res. Commun..

[40] Tang Y., Bao J.S., Su J.H., Huang W. (2017). MicroRNA-139 modulates Alzheimer’s-associated pathogenesis in SAMP8 mice by targeting cannabinoid receptor type 2. Genet. Mol. Res..

[41] O’Sullivan S.E. (2016). An update on PPAR activation by cannabinoids. Br. J. Pharmacol..

[42] Turu G., Hunyady L. (2010). Signal transduction of the CB1 cannabinoid receptor. J. Mol. Endocrinol..

[43] Bosier B., Lambert D.M., Hermans E. (2008). Reciprocal influences of CB1cannabinoid receptor agonists on ERK and JNK signalling in N1E-115 cells. FEBS Lett..

[44] Idris A.I., van’t Hof R.J., Greig I.R., Ridge S.A., Baker D., Ross R.A., Ralston S.H. (2005). Regulation of bone mass, bone loss and osteoclast activity by cannabinoid receptors. Nat. Med..

[45] Wang F.S., Wang C.J., Chen Y.J., Chang P.R., Huang Y.T., Sun Y.C., Huang H.C., Yang Y.J., Yang K.D. (2004). Ras induction of superoxide activates ERK-dependent angiogenic transcription factor HIF-1alpha and VEGF-A expression in shock wave-stimulated osteoblasts. J. Biol. Chem..

[46] Kohda Y., Murakami H., Moe O.W., Star R.A. (2000). Analysis of segmental renal gene expression by laser capture microdissection. Kidney Int..

[47] Lin C.L., Wang J.Y., Ko J.Y., Huang Y.T., Kuo Y.H., Wang F.S. (2010). Dickkopf-1 promotes hyperglycemia-induced accumulation of mesangial matrix and renal dysfunction. J. Am. Soc. Nephrol..

